# Similar Expectation Effects for Immediate and Delayed Stimulus Repetitions

**DOI:** 10.3389/fnins.2019.01379

**Published:** 2019-12-20

**Authors:** Catarina Amado, Sophie-Marie Rostalski, Mareike Grotheer, Nadine Wanke, Gyula Kovács

**Affiliations:** ^1^Experimental Cognitive Science, Eberhard Karls Universität Tübingen, Tübingen, Germany; ^2^Biological Psychology and Cognitive Neurosciences, Institute of Psychology, Friedrich Schiller University Jena, Jena, Germany; ^3^Department of Psychology, Stanford University, Stanford, CA, United States; ^4^Institute of Psychology, University of Hamburg, Hamburg, Germany

**Keywords:** expectation, fMRI adaptation, prediction, repetition suppression, inter-stimulus interval

## Abstract

A prior cue or stimulus allows prediction of the future occurrence of an event and therefore reduces the associated neural activity in several cortical areas. This phenomenon is labeled expectation suppression (ES) and has recently been shown to be independent of the generally observed effects of stimulus repetitions (repetition suppression, RS: reduced neuronal response after the repetition of a given stimulus). While it has been shown that attentional cueing is strongly affected by the length of the cue-target delay, we have no information on the temporal dynamics of expectation effects, as in most prior studies of ES the delay between the predictive cue and the target (i.e., the inter-stimulus interval, ISI) was in the range of a few hundred milliseconds. Hence, we presented participants with pairs of faces where the first face could be used to build expectations regarding the second one, in the sense that one gender indicated repetition of the same face while the other gender predicted the occurrence of novel faces. In addition, we presented the stimulus pairs with two different ISIs (0.5 s for Immediate and 1.75 or 3.75 s for Delayed ISIs). We found significant RS as well as a reduced response for correctly predicted when compared to surprising trials in the fusiform face area. Importantly, the effects of repetition and expectation were both independent of the length of the ISI period. This implies that Immediate and Delayed cue-target stimulus arrangements lead to similar expectation effects in the face sensitive-visual cortex.

## Introduction

Repetition related phenomena have been widely studied using both electrophysiological and neuroimaging techniques. Typically these studies report suppression of the neural signal for repeated when compared to alternating stimuli (repetition suppression, RS; Henson and Rugg, 2003; for review see Grill-Spector et al., 2006). RS has been explained in many ways (i.e., synaptic depression, network dynamics, and facilitation of the neural response) and has become one of the most intensively studied phenomena in cognitive neurosciences. Further, it is broadly applied as a tool to investigate the selective properties of neuronal populations in neuroimaging experiments (fMRI adaptation; Malach, 2012).

Recently, the neural mechanisms of RS have been connected to predictive coding theories of sensory perception (PC, see Friston, 2005; Auksztulewicz and Friston, 2016). According to models of PC, the brain constantly generates predictions about sensory inputs and then computes the difference between these predictions and the actual sensory input. Therefore, surprising/incorrectly predicted events cause higher neural activity than expected/correctly predicted events (Friston, 2005, 2010; Friston and Kiebel, 2009). In other words, the occurrence of an expected event can also lead to reduced neuronal activity when compared to incorrect predictions, i.e., to surprising events. This phenomenon was recently labeled expectation suppression (ES, Todorovic and de Lange, 2012).

In an influential study, Summerfield et al. (2008) presented participants with pairs of faces that could either repeat or alternate. These faces were grouped into blocks with either high (75%, RB) or low (25%, AB) repetition probabilities (P(rep)]. The results revealed larger RS in the fusiform face area (FFA; Kanwisher et al., 1997) in blocks with more repetitions (RB), and hence more expected when compared to blocks with fewer repetitions, and thus surprising repetitions (AB). Therefore, the authors suggested that higher-order contextual expectations modulated repetition-related processes. Other studies confirmed the existence of such P(rep) modulations of RS for faces (Kovács et al., 2012, 2013; Larsson and Smith, 2012; Grotheer et al., 2014) and Roman letters (Grotheer and Kovács, 2014). While no such modulations were found for chairs (Kovács et al., 2013) or unfamiliar characters (Grotheer and Kovács, 2014), but for a different conclusion see Mayrhauser et al. (2014). All of these studies used a factorial design in which repetition and repetition probability varied orthogonally. However, they did not allow the independent testing of expectation and repetition effects due to the use of high and low repetition blocks to manipulate expectations.

Other studies have induced explicit perceptual expectations on a trial-by-trial basis by associating a given stimulus with a preceding schematic cue or image (Egner et al., 2010; Meyer and Olson, 2011). Current MEG and neuroimaging studies have combined such paradigms with stimulus repetitions, in the sense that the first stimulus of a pair signals the likelihood of repetitions or alternations, and found both ES and RS to be present in the target-related signal (Todorovic and de Lange, 2012; Grotheer and Kovács, 2015; Amado and Kovács, 2016). Importantly, both the MEG and the neuroimaging studies have found that the effects of expectation and repetition are independent and additive processes in the human brain. Moreover, a recent EEG study (Feuerriegel et al., 2018) also investigated whether repetition effects are influenced by perceptual expectations and found distinct spatiotemporal patterns of repetition and expectation effects, supporting the idea of separable mechanisms underlying these phenomena.

Earlier studies have explored the influence of the inter-stimulus interval (ISI) length on RS and showed similarities between short and long-lagged repetition effects (Henson et al., 2004; Sayres and Grill-Spector, 2006), but it has also been suggested that different neuronal mechanisms explain RS for long and short ISIs (Epstein et al., 2008; Kouider et al., 2009; Weiner et al., 2010; Larsson and Smith, 2012). Additionally, both electrophysiological (Feuerriegel et al., 2015) and behavioral (Matthews, 2015) studies of RS and repetition priming, describing behavioral response improvements for repeatedly presented stimuli, have reported distinct effects of stimulus duration and ISI variability.

Moreover, it is also known that ISI length affects attentional cueing (Hansen and Hillyard, 1980; Busse et al., 2006). Briefly, attentional cueing experiments rely on the flexible allocation of attention to specific aspects of the sensory stimulation, such as certain features of the stimuli, as well as their temporal or spatial properties. In general, attention can be driven both by top-down (i.e., cognitive expectations, called “endogenous” attention) or bottom-up (i.e., sensory events, called “exogenous” attention) processes (Hopfinger and West, 2006). The nature of the cue determines the type of attentional process (see Posner and Cohen, 1984). Interestingly, the ISI length seems to interfere with exogenous and endogenous attention in a different manner. At short durations (at around 2 s), endogenous attention enhances perceptual sensitivity (through an improvement in the accuracy of the responses). However, at longer durations (typically larger than 4 s) endogenous attention can actually impair stimulus sensitivity (Ling and Carrasco, 2006). In the case of exogenous attentional processes, the responses are faster and more accurate when valid cues are presented with short intervals between the cue and the target. If, however, the ISI length is large the participants’ reactions for valid cues will be slower (i.e., larger than 300 ms; see Posner and Cohen, 1984) and less accurate (Handy et al., 1999) than for invalid cues. Also, Busse et al. (2006) found facilitation of the behavioral response (in terms of shorter RTs) with short cue-target ISIs, only when both location and feature cues were valid. Longer ISIs induced the opposite effect, as the RTs were longer when the targets appeared at the cued location.

In terms of the PC theory expectations are probability-based top-down information that are tested against sensory input. Endogenous attention can be connected to the term perceptual expectation as both can rely on cues on a trial-by-trial basis (Meyer and Olson, 2011). In spite of the demonstrated effects of ISI on RS and on attentional cueing, previous studies which have investigated ES have invariably used short (in the range of few hundred milliseconds) delay-intervals between the predictive cue and the target (Egner et al., 2010; Grotheer and Kovács, 2015; Amado and Kovács, 2016).

Since we have no information on the temporal dynamics of cue-based expectation effects (Matthews and Gheorghiu, 2016), the current study aimed to investigate whether additive effects of RS and ES are consistent across changes of the presentation delay. To this end, we used the methods, task, and paradigm of Grotheer and Kovács (2015) with different ISI lengths. To anticipate our results, we observed significant RS and ES in the FFA, but we did not find any interaction between ES and RS for either ISI conditions, suggesting that the length of ISI does not influence the neural mechanisms of ES and RS.

## Materials and Methods

### Participants

Twenty-six healthy Caucasian volunteers participated in the experiment. The number of participants was chosen based on our prior published works. In our previous papers testing RS (Grotheer et al., 2014: *n* = 26; Grotheer and Kovács, 2014: *n* = 17; Grotheer and Kovács, 2015: *n* = 25; Amado et al., 2016: *n* = 22; Hermann et al., 2016: *n* = 29) we invariably tested similar number of participants and observed always strong and reliable RS as well as probability-based modulations of RS. Therefore, we did not use any specific way to estimate the sample sizes here. No participant reported any neurological or psychiatric illnesses and all subjects had normal or corrected to normal visual acuity and gave written informed consent in accordance with the protocols approved by the Ethical Committee of the Friedrich-Schiller-University Jena by following the Declaration of Helsinki. Overall, three participants were excluded from the final analysis. One was excluded due to excessive head-movements (i.e., translation/rotation of > 5 mm/degrees) during the recording, while another participant failed to perform the experimental task properly (the performance was below 50% in one experimental run) and one participant interrupted the recording session. Therefore, the current report is based on the data of 23 participants (17 females; 20 right-handed, mean age (±SD): 21.6 (0.7) years).

### Stimulation and Procedure

**FIGURE 1:**
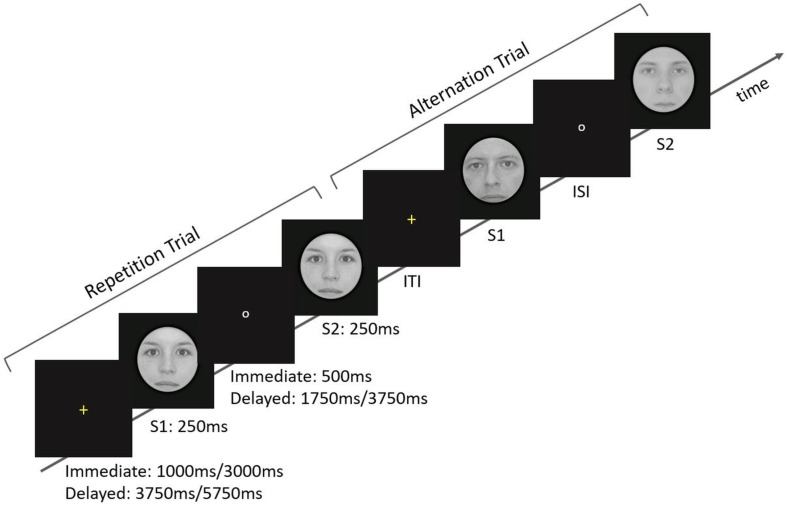
Overview of the stimulation parameters and arrangements. At the beginning of each trial, a yellow fixation cross was presented for 1 or 3 s in the *Immediate* ISI condition and for 3.75 or 5.75 s in the *Delayed* ISI condition. The cross was followed by the predictive cue, S1, which was shown for 250 ms. During the ISI a small white circle appeared on the screen. The ISI conditions correspond to *Immediate* and *Delayed* lengths of fix 500 ms and varying 1.75/3.75 s, respectively. Finally, the target, S2, was presented for 250 ms. Note that *Immediate* and *Delayed* trials were given in separate runs.

Stimuli were 300 gray-scale, digital photos of full-frontal Caucasian faces (2.75° visual angle), identical to those of Grotheer and Kovács (2015). Briefly, stimuli were fit behind a circular mask, placed in the center of the screen on a uniform black background. Stimulus pairs were presented, with 250 ms presentation time for each stimulus. We only used Caucasian faces as it is known that the own-race bias results in differences regarding the perceptual expertise with own when compared to other-race faces (for review see: Meissner and Brigham, 2001). Two ISI conditions were used. In the *Immediate* condition, the ISI was 500 ms, and hence identical to that of previous publications (Grotheer and Kovács, 2015; Amado and Kovács, 2016). In the *Delayed* condition, the ISI was varied randomly between 1.75 and 3.75 s (this temporal jitter was introduced to help the separation of the BOLD response, related to S1 and S2 as these two are not presented within one TR anymore). The two ISI trial types (*Immediate* and *Delayed*) were presented in two separate runs in an order randomized across participants. The inter-trial intervals were randomized between 1 and 3 s or between 3.75 and 5.75 s for the *Immediate* and *Delayed* conditions, respectively (see Figure 1). This relatively short time-range for the *Delayed* ISI condition was chosen because the further elongation of the ISI (to the order of minutes) would have led to an experiment-duration up to 2 h. Two runs were recorded from each participant (one for each ISI condition) and no stimulus occurred in more than one trial during a given run (i.e., the same stimulus could occur in two different runs). The runs contained 180 trials and lasted for about 11 and 25 min for the *Immediate* and *Delayed* conditions, respectively. Stimuli were back-projected via an LCD video projector (NEC GT 1150, NEC Deutschland GmbH, Ismaning, Germany, with modified lens for short focal point) onto a translucent circular screen, placed inside the scanner bore [stimulus presentation was controlled by Matlab R2013a (The MathWorks, Natick, MA, United States), using Psychtoolbox (Version 3.0.9)].

Trial structure and design were identical to those of Grotheer and Kovács (2015) and Amado and Kovács (2016). We used a paired stimulus presentation where the predictive cue, the first stimulus (S1), could either be different [Alternation Trial (Alt)] or identical [Repetition Trial (Rep)] to the second, target stimulus (S2). To reduce local feature adaptation the size of either S1 or S2 (chosen randomly) was reduced by 18%. Both stimuli of a pair were either female or male and participants were presented with 50% female/male trials randomly. The gender of S1 cued stimulus repetition or alternation to the participants probabilistically, meaning high (75%) or low (25%) probabilities of repetition/alternation of the target stimulus (S2). For example, for half of the subjects, female faces signaled high repetition probability (75%), while male faces signaled high alternation probability (75%). This way, participants could form expectations regarding the likelihood of repetitions and alternations. Correctly predicted trials correspond to a congruence between the given cue (S1) and the repetition/alternation occurrence during S2 (75% of the trials), whereas the incorrectly predicted, or surprising trials correspond to an incongruency between the given cue (S1) and the repetition/alternation occurrence during S2 (25% of the trials). The relationship between face gender and repetition probability was counterbalanced across participants (11 participants in one version and 12 in the other one), in a way that for the other half of the subjects (*N* = 11) the gender cueing high repetition probability was male and the relative repetition probabilities were reversed accordingly. Participants were informed about the relative repetition/alternation probabilities as well as about their contingencies on the face gender of S1 prior to the scanning sessions. In addition, participants performed a 5-min long training session (using stimuli that were different from those used in the main experiment) immediately prior to the fMRI recordings.

Briefly, a trial started with a yellow fixation cross, which was presented for 1 or 3 s in the *Immediate* ISI condition and 3.75 or 5.75 s in the *Delayed* ISI condition. Participants were asked to fixate it. The cross was followed by the predictive cue, S1, which was shown for 250 ms. During the ISI a small white circle appeared on the screen. The ISI conditions correspond to *Immediate* and *Delayed* lengths of fix 500 ms and varying 1.75/3.75 s, respectively. Finally, the target, S2, was presented for 250 ms.

Moreover, following the method of Larsson and Smith (2012), 20 (11.1% of the trials) additional blank trials were included in each run to enable the estimation of the average response time course to the target stimulus (S2) alone. In these trials, S1 was normally displayed and instead of S2, a blank screen was presented. This way, an estimate of the average response time course to S2 alone was obtained by performing a subtraction between the blank trials and the experimental conditions which included S2 and S1 as well. In order not to bias the predictions of participants, these trials had an equal amount of female and male faces for S1. Importantly, the overall probabilities for the correctly predicted and surprising conditions were 66.7 and 22.2%, respectively. As the introduction of the blank trials made the separation of subsequent trials perceptually more difficult, the color of the fixation cross was changed to yellow before the presentation of S1, to clearly mark the beginning of trials.

**FIGURE 2:**
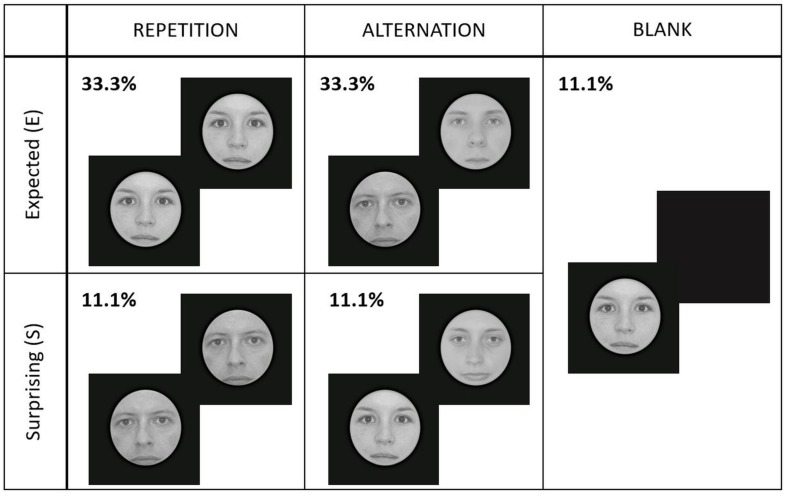
Experimental design and conditions. Each face gender signaled different repetition/alternation probabilities (high or low) randomly for every participant. Here we present an example where the face gender signaling high repetition probability was female (E_Rep), while male faces cued high probability of alternations (E_Alt). Male/female faces signaled low probability of repetitions/alternations (S_Rep/S_Alt). Blank trials contained either female or male faces, randomly.

In total, we had five different experimental conditions, presented randomly within a run: expected repetition (E_Rep), expected alternation (E_Alt), surprising repetition (S_Rep), surprising alternation (S_Alt), and blank (Blank) trials. Figure 2 illustrates the experimental design.

To control participants’ attention and to confirm that they are able to judge the stimulus gender effectively, 18% of the trials were target trials in which subjects had to report whether the S1 had been a female or male face by pressing a button (Todorovic and de Lange, 2012). Therefore, for these target trials, a choice-screen was presented for 2 s centrally showing either the text “*female? male*” or “*male? female*,” randomly. The choice-screen appeared 1 s after S2 was blanked out. A small color change of the fixation cross functioned as feedback regarding their answers (green for correct and red for incorrect responses).

### Imaging Parameters and Data Analysis

Imaging was done with a 3-Tesla MR scanner (Siemens MAGNETOM Prisma fit, Erlangen, Germany). T2^∗^ weighted images were collected using an EPI sequence (35 slices, 10° tilted relative to axial, TR = 2000 ms; TE = 30 ms; flip angle = 90°; 64 × 64 matrices; 3 mm isotropic voxel size). A high-resolution T1-weighted 3D anatomical image was acquired using an MP-RAGE sequence (TR = 2300 ms; TE = 3.03 ms; 192 slices; 1 mm isotropic voxel size).

Details of preprocessing and statistical analysis were described previously (Cziraki et al., 2010). The functional images were realigned, normalized to the MNI-152 space, resampled to 2 × 2 × 2 mm resolution and spatially smoothed with a Gaussian kernel of 8 mm FWHM (SPM12, Wellcome Department of Imaging Neuroscience, London, United Kingdom). A separate functional localizer run (640 s long, 20-s epochs of faces, objects and Fourier randomized versions of faces, interleaved with 10 s of blank periods, 2 Hz stimulus repetition rate; 300 ms exposure; 200 ms blank) served as a basis for Regions of Interest (ROIs) detection. ROI creation was performed with MARSBAR 0.44 toolbox for SPM (Brett et al., 2002). Only those individuals in whom the respective ROIs could be identified in both hemispheres were included in the further analyses. The FFA was determined individually as an area responding more intensely to faces than to objects and Fourier randomized versions of faces (*p* < 0.0001_UNCORRECTED_). Its location could be identified reliably and bilaterally in 20 participants [average MNI coordinates (±SE): 41 (0.6), −54 (1.3), −19 (0.8), and −41 (1.4), −57 (1.7), −18 (0.7); average cluster size (±SE): 72(7), 52(5) voxels; for the right and left hemispheres, respectively].

A time series of the mean voxel value within the areas of interest was calculated and extracted from our event-related sessions using custom made scripts and Marsbar. The convolution of each of the five experimental conditions (E_Rep, E_Alt, S_Rep, S_Alt, Blank) with the canonical hemodynamic response function (HRF) of SPM12 (Welcome Department of Imaging Neuroscience, London, United Kingdom) was used to define predictors for a General Linear Model (GLM) analysis of the data. Target trials were not modeled separately, as there was sufficient time (1 s) between the end of the trial and the choice-screen presentation. Thus, the BOLD signal of the S2 was not affected by the button presses or by the choice-screens. Note that the subtraction between blank trials and the other experimental conditions (E_Rep, E_Alt, S_Rep, S_Alt) was executed to estimate the average response time course to S2 alone (Larsson and Smith, 2012). The peak values of the BOLD signal elicited by S2 only were submitted to the following statistical analysis. We performed repeated measures ANOVAs for the FFA activity separately with hemisphere (2), expectation level (2, E and S), trial type (2, Alt and Rep) and ISI condition (2, Immediate and Delayed) as factors. *Post hoc* analyses were executed using Fisher LSD tests. We also performed a *t*-test and calculated Bayes factor (e.g., Dienes, 2011) to test the independence of RS/ES from the ISI length and denoted evidence according to the thresholds proposed by Jeffreys (1940). We used the following prior hypothesis: RS and ES effects are larger in the Immediate ISI condition than in the Delayed one, therefore the reported results show how much more likely our hypothesis is when compared with the null hypothesis. In order to perform a *t*-test and directly compare the effects of repetition and expectation suppression for the two ISI conditions, we calculated the repetition suppression index (RSI = Alt-Rep) and the expectation suppression index (ESI = Sur-Exp).

As there is evidence that prediction error units of FFA can be activated by a positive prediction error (i.e., the occurrence of an unexpected face), but not by a negative one (i.e., the unexpected omission of a face; see Egner et al., 2010). We decided to test the influence of stimulus omission in this experiment by performing a repeated measures ANOVAs for the FFA activity separately with ISI condition (2, Immediate and Delayed) and omission level (2, Blanked and Non-blanked trials) as factors. *Post hoc* analyses were executed using Fisher LSD tests.

## Results

### Behavior

Participants required on average 981 ms (±SD: 45 ms) to determine the gender of the presented S1 faces. Reaction times did not differ significantly between trial types (*F*(1,22) = 1.15, *p* = 0.29, η*_*p*_*^2^ = 0.05), expectation levels (*F*(1,22) = 0.24, *p* = 0.63, η*_*p*_*^2^ = 0.01) or ISI conditions (*F*(1,22) = 2.22, *p* = 0.15, η*_*p*_*^2^ = 0.09). Similarly, only tendencies were observed for any of the interactions (*p* > 0.08 for all comparisons). We found a tendency for an interaction between expectation levels and ISI conditions (*F*(1,22) = 3.18, *p* = 0.088, η*_*p*_*^2^ = 0.126), showing that correctly predicted trials differed between ISI conditions [being faster for *Immediate* trials (*M*(±*SD*) = 927 (39)ms) as compared to *Delayed* ones (*M*(±*SD*) = 1018 (35)ms), *p* = 0.003], while incorrect predictions did not show any difference.

Mean accuracy for gender judgment was 86% (±SD: 3%) across all experimental conditions. The participants’ accuracies did not differ between trial types (*F*(1,22) = 1.53, *p* = 0.22, η*_*p*_*^2^ = 0.07) and ISI conditions (*F*(1,22) = 1.62, *p* = 0.22, η*_*p*_*^2^ = 0.07). Further, no significant interactions were observed (*p* > 0.08 for all comparisons). Interestingly, and confirming previous results (Grotheer and Kovács, 2015; Amado and Kovács, 2016), there was weak evidence for a main effect of expectation level (*F*(1,22) = 3.4, *p* = 0.08, η*_*p*_*^2^ = 0.13), showing an enhanced accuracy for correctly predicted (*M*(±*SD*) = 88 (3)%) when compared to surprising (*M*(±*SD*) = 82 (5)%) trials.

The similar accuracy rates and response times suggest a similar allocation of attention to the different experimental conditions.

### Fusiform Face Area

**FIGURE 3:**
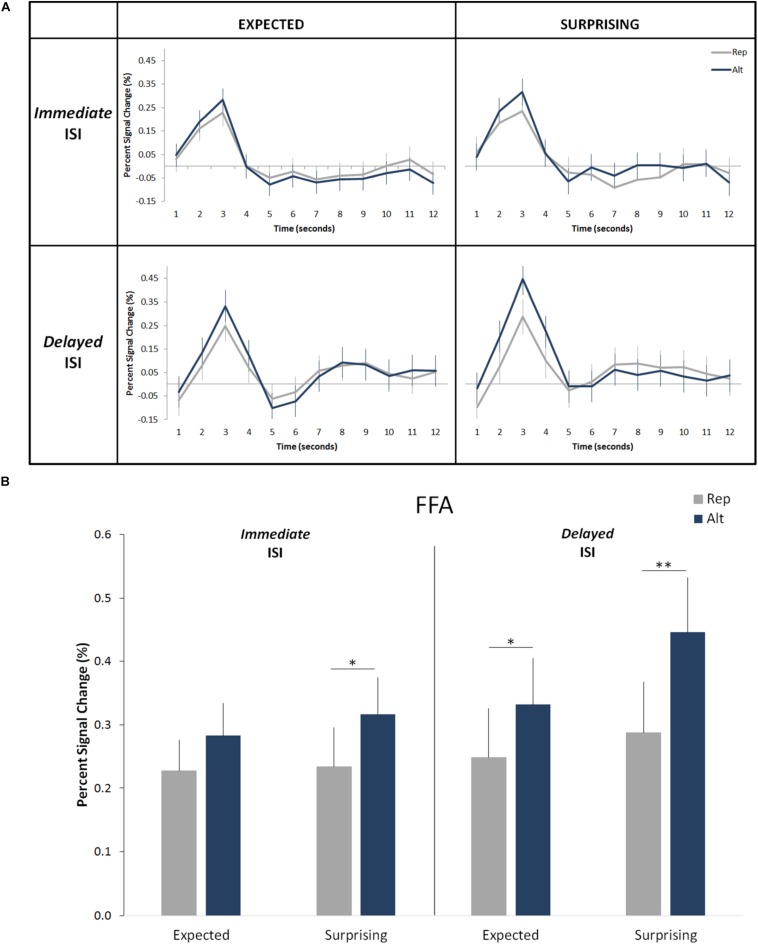
Effects of expectation and repetition on the FFA responses (averaged left and right hemispheres) for different ISI conditions. **(A)** Average response time course for Rep and Alt trials in expected (correctly predicted; left) and surprising (incorrectly predicted; right) events for the *Immediate* (up) and *Delayed* (down) ISIs. **(B)** Percent-signal changes (±SE) are presented separately for trials types, expectation levels and ISI conditions. ^∗∗^*p* < 0.001; ^∗^*p* < 0.05.

Overall, the results confirmed those of our prior studies (Grotheer and Kovács, 2015; Amado and Kovács, 2016). We observed a significant main effect of trial type (i.e., significant RS; Figure 3; *F*(1,19) = 25.09, *p* = 0.0008, η*_*p*_*^2^ = 0.57) with an average signal reduction of 0.1% (equivalent to an average relative signal reduction of 27%). We also found a main effect of expectation level (i.e., significantly higher responses for surprising as compared to correctly predicted events: *F*(1,19) = 5.65, *p* = 0.028, η*_*p*_*^2^ = 0.23). On average the correct predictions led to a signal reduction of 0.05% (corresponding to an average relative signal decrease of 16%) when compared to the incorrect predictions. No main effect of hemisphere was found (*F*(1,19) = 1.27, *p* = 0.27, η*_*p*_*^2^ = 0.06). Additionally, the effects of trial type and expectation level did not interact with each other (*F*(1,19) = 3.08, *p* = 0.10, η*_*p*_*^2^ = 0.14), but were additive (Figure 3).

More important to the aims of the current study, we did not find a significant main effect of ISI condition (*F*(1,19) = 1.68, *p* = 0.21, η*_*p*_*^2^ = 0.08). There was neither an interaction of ISI condition with the effect of trial type (*F*(1,19) = 0.37, *p* = 0.54, η*_*p*_*^2^ = 0.02) nor with the effect of expectation (*F*(1,19) = 1.2, *p* = 0.28, η*_*p*_*^2^ = 0.06). The four-way interaction of the hemisphere, ISI condition, trial type and expectation was not significant either (*F*(1,19) = 0.53, *p* = 0.48, η_*p*_^2^ = 0.03). None of the remaining two-way and three-way interactions are significant. This suggests that both RS and ES are independent of the length of the ISI period. The Bayesian *t*-test revealed that both effects of neuronal response suppression, i.e., RS (B_10_ < 0.2) and ES (B_10_ < 0.2) are independent of the ISI length.

We found a significant main effect of omission level [i.e., larger BOLD responses to the non-blank trials when compared to blank trials; *F*(1,19) = 53.95, *p* = 0.000001, η*_*p*_*^2^ = 0.59]. No interaction was found between the ISI condition and the omission level (*F*(1,19) = 1.44, *p* = 0.23, η*_*p*_*^2^ = 0.074).

Importantly, the two ISI conditions of this study differ in terms of ISI variability characteristics and predictability. Although we included blank trials in both ISI condition blocks, in the *Immediate* ISI condition, there is only one possible ISI length (500 ms), and therefore the stimulus onset is nearly fully predictable in time. While, in the *Delayed* ISI condition, there are two possible ISIs (long – 5.75 s and short – 3.75 s). In this condition, the longer one is nearly fully predictable, as it will occur whenever there was no stimulus after 3.75 s and the current trial isn’t a blank trial. The short ISI in the *Delayed* condition is only expected in 44, 45% of the trials. To test whether the results were affected by these differences in the variability and predictability characteristics of the ISI length of the *Immediate* (constant and fully predictable) and the *Delayed* ISI condition (variable and semi-predictable, i.e., long – 5.75 s and short – 3.75 s), we performed a repeated measures ANOVA to compare the BOLD responses of the two fully predictable conditions, i.e., the longer *Delayed* ISI lengths and the *Immediate* ISI condition. This extra analysis only revealed to be significant in two main effects: repetition suppression (*F*(1,19) = 15.63; *p* = 0.0009; η*_*p*_*^2^ = 0.45) and ISI (in a way that the *Immediate* ISI length elicited larger BOLD responses when compared with the longer *Delayed* ISI condition; *F*(1,19) = 18.88; *p* = 0.0004; η*_*p*_*^2^ = 0.47).

### Whole-Brain Analysis

**FIGURE 4:**
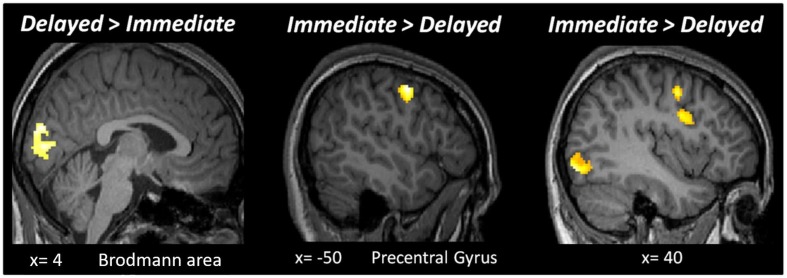
Results of the whole-brain analysis with a fixed threshold of *p* < 0.05_FWE_, with a cluster size bigger than 50 voxels for the following contrasts: *Delayed* > *Immediate* and *Immediate* > *Delayed*.

It is theoretically possible that expectation and repetition effects are encoded elsewhere in the brain. Hence, we performed a second level whole-brain analysis testing for the main effects of RS, ES, ISI and the interaction of these factors, using a fixed threshold of *p* < 0.05_FWE_, with a cluster size > 50 voxels. Testing the main effect of ISI (*Delayed* > *Immediate*) revealed an active cluster in the early visual cortex (MNI [*x*,*y*,*z*]: 4, −86, 20; cluster size: 288), while the opposite contrast (*Immediate* > *Delayed*) led to several regions with significant activations (Figure 4). The whole-brain analysis did not reveal additional active clusters when testing for the main effects of RS and ES or the interactions between ES, ISI, and RS.

In order not to overlook any activation on the whole-brain level (however, see the recent discussion, initiated by Eklund et al. (2016) about the inflated false-positive rates of such cluster analyses) we also applied a more liberal *p* < 0.0001_uncorrected_ threshold with a smaller cluster size (>20 voxels). Both the *Immediate* > *Delayed* and the *Delayed* > *Immediate* contrasts showed some additional regions with significant activations (Table 1). Yet, once again, no additional active clusters were found when testing for the main effects of RS and ES or for the interactions between ES, ISI and RS, supporting the results of the ROI analysis. In principle, one would expect the FFA to be activated in the whole-brain analysis when testing for the main effects of RS and ES as well. Still, it is likely that the lower sensitivity of the whole-brain, when compared to the ROI based analysis (Nieto-Castanon et al., 2003), as well as the large inter-individual difference in the peak location of the FFA (Zhen et al., 2015) accounts for the lack of such an observation.

**TABLE 1:** Summary of significant activations in the whole-brain analysis.

**Contrast**	**Brain region**	**Coordinates**	**Cluster size**	**Threshold**
*Delayed* > *Immediate*	Brodmann area 18	4, −86, 20	288	(*p* < 0.05 FWE)
*Delayed* > *Immediate*	Inter-Hemispheric	0, −62, 56	57	(*p* < 0.0001 unc)
*Delayed* > *Immediate*	Brodmann area 6	56, −4, 6	23	(*p* < 0.0001 unc)
*Immediate* > *Delayed*	Precentral Gyrus	−50, −4, 48	241	(*p* < 0.05 FWE)
*Immediate* > *Delayed*	Inferior Occipital Gyrus	40, −84, −10	405	(*p* < 0.05 FWE)
*Immediate* > *Delayed*	Brodmann area 6	50, 2, 48	481	(*p* < 0.05 FWE)
		6, 8, 54	143	(*p* < 0.05 FWE)
*Immediate* > *Delayed*	Sub-Gyral	28, −50, 46	67	(*p* < 0.05 FWE)
*Immediate* > *Delayed*	Lingual Gyrus	−18, −88, −8	211	(*p* < 0.05 FWE)
*Immediate* > *Delayed*	Middle Frontal Gyrus	−28, −5, 54	61	(*p* < 0.0001 unc)

## Discussion

We observed significant repetition and expectation effects in the FFA in the form of reduced responses for repeated and expected stimuli, respectively. These effects were found to be additive and independent of the length of ISI and imply that *Immediate* and *Delayed* predictive cueing produce similar effects of expectation related response suppression in the FFA, suggesting that the observed expectation effects survive a several second-long time-interval. The fact that RS and ES were found to be additive and thereby independent from each other for both ISI lengths confirms the results of recent studies that used short ISIs (Todorovic and de Lange, 2012; Grotheer and Kovács, 2015; Amado et al., 2016).

### Repetition Suppression

Earlier RS studies, using different ISI lengths, have suggested that RS is stable over short cue-target periods (in the range of 250 ms to 4 s) for object stimuli in an fMRI experiment (Henson et al., 2004; Sayres and Grill-Spector, 2006), which is in accordance with our results showing no difference in RS across ISI lengths. However, if ISIs are prolonged further (maximum of 8 min) several studies propose that the neural mechanisms underlying RS with short ISIs (less than 3 s) are different from those underlying RS with long ISIs (Henson et al., 2004; Sayres and Grill-Spector, 2006).

For example, Epstein et al. (2008) reported that the effect of ISI on RS for visual scenes measured in the fMRI depends on scene viewpoint (in the range of 500 ms to 8 min, for short and long ISIs, respectively), in other words, short-interval RS was only significant when scenes were repeated from the same viewpoint, while long-interval RS was less viewpoint-dependent. Also, long- and short-interval RS effects did not interact at all. Furthermore, Weiner et al. (2010) used objects as stimuli and showed that RS varies quantitatively across time periods in the ventral temporal cortex. This study used ISI categories which are somewhat different from those used in the current study: the short and the long ISI periods were 500 ms to 3 s and of 1 to 174 s, respectively. Therefore, in the study of Weiner et al. (2010) there was an overlap of durations in the short and long ISI conditions, which was not present in the current study. Additionally, the maximum length of their “short” ISI is comparable to our *Delayed* condition and they did not study RS on a trial by trial basis. Please note that Weiner et al. (2010), as well as Epstein et al. (2008) and Henson et al. (2004) used object stimuli and therefore also tested different regions. All these facts make the comparison to the current study difficult.

Face studies have found that with long ISIs (in the range of 7 min), the effects of repetition depend on familiarity such that RS only occurs for familiar faces (Kouider et al., 2009). In this study, participants had to judge face familiarity. The results revealed that face-processing occurs even without perceptual awareness. Furthermore, different face viewpoints were also investigated for the short-lagged (subliminal priming) condition, yet no effects of viewpoint were found for either the familiar or unfamiliar faces. Note that the minimum duration for the long-lagged condition was 7 min in their study, which is considerably larger than the 3.75 s applied in the current study. Importantly, instead of a blank screen, in this study, a mask was presented between S1 and S2 to manipulate perceptual awareness. The use of shorter lengths and the absence of this mask in the ISI period might explain why we found RS effects with unfamiliar faces for the *Delayed* condition as well. Also, the current study did not include familiarity as a factor. It will be important to determine what role familiarity plays in expectations and RS with specifically designed future experiments that are comparable to those of the study of Kouider et al. (2009). Another study using face stimuli and examining the impact of different cue-target intervals is from Larsson and Smith (2012). They investigated how probability-based expectations affect RS with longer ISIs and showed that P(rep) modulation of RS exists with longer (4 s) cue-target periods but that this effect depends on attention. These findings are in accordance with our results, despite the fact that Larsson and Smith (2012) induced expectations implicitly, based on the differential probabilities of trials within blocks, while here expectations were manipulated explicitly, with a cue, on a trial-by-trial basis. Please note, that the main goal in their study was to show the effect of attention on probability-based RS modulation.

A recent electrophysiological study has investigated not only how RS varies with different ISI periods but also how it is influenced by diverse stimulus presentation durations of S1 (Feuerriegel et al., 2015). Their results indicate no effect of ISI period on the N170 amplitudes for faces or chairs. However, the amplitude of the positive P2 component was lowest when the ISI was short (200 ms). As is known, electroencephalography has better temporal resolution than fMRI, and this fact can possibly explain incongruences between that and the current studies. Also, the ISI periods of this study varied from 200 to 500 ms, which is in the range of our *Immediate* condition and makes comparison difficult. Anyway, further electrophysiological studies are also necessary to evaluate how expectation effects modulate RS in different cue-target stimulation periods.

### Expectation

Notably, no ISI effects on ES were observed on the behavioral data or on the BOLD signal in the current study, whereas Busse et al. (2006) reported reaction time facilitation for expected events that were presented with short cue-target stimulus periods. In other words, if expectations are fulfilled (the target can be predicted) and the cue and the target appear in a narrow time window response times are shortened (which fits the predictive coding framework). The discrepant results of Busse et al. (2006) and our current study can easily be explained by the lower number of trials in our study [360 vs. 500 in Busse et al. (2006)] and the application of different stimuli (moving dots vs. faces). Also, Busse et al. (2006) used an exogenous cue, whereas we applied endogenous cues, signaling the appearance of subsequent images.

Another behavioral study inspected how time perception depends on different durations of stimulus presentation and ISI (Matthews, 2015). Following the paradigm of previous studies (Summerfield et al., 2008; Larsson and Smith, 2012), this behavioral study used the probabilities of repetitions in each block to manipulate expectations. Interestingly, repeats were judged longer than novel items for shorter ISIs, but this effect was more pronounced when the repetitions were rare. For the longer ISI condition repeated and novel images were judged the same.

The fact that we found similar ES for the *Immediate* and *Delayed* conditions is in line with theories of predictive coding (Friston and Kiebel, 2009, PC). PC explains the brain as a cascadic system of parallel feed-forward and feedback processes in which the sensory information is continuously compared to the current expectations of the system, based on prior experiences, and only the difference of the two, the predictive error, is propagated to higher-level areas (Friston, 2010). The predictive error is calculated and updated continuously in such a system. Whether there is an upper time-limit of the influence of the predictive stimuli is still an open question. Our results, however, suggest that the effect of the calculated predictions is not only manifest for immediate subsequent phenomena but also extends to a time range of several seconds, increasing the stability of the system. Recently, the processing of sensory information and most of the neuronal phenomena, such as RS and ES is explained under the predictive coding framework more and more widely. This framework assumes that, for example, visual processing occurs in a hierarchical manner in which lower-level areas receive predictions about the incoming sensory input from higher-order areas through feedback connections (Friston, 2005). Consequently, when the sensory input coincides with the created high-level expectations, there is a suppression of the predicted neural responses in lower level areas, due to an inhibited response of these neuronal populations in the form of an efficient encoding mechanism (Friston and Kiebel, 2009).

Given the universal nature of PC, it is rather surprising that some recent findings disagree with the PC explanations of the neuronal response suppression. Evidence comes from studies that used non-face stimuli (fractals and chairs) and found no repetition probability modulations of RS in macaques’ inferior temporal (Kaliukhovich and Vogels, 2011) and humans’ lateral occipital cortices (Kovács et al., 2013) (but see Mayrhauser et al., 2014 for a different conclusion). These results are in contrast to what had been found for faces and voices, i.e., a strong modulation of RS by P(rep) (Summerfield et al., 2008; Kovács et al., 2012; Larsson and Smith, 2012; Andics et al., 2013). Therefore, the question if the observed similarity of short and long-term ISI on P(rep) in our study is a general property of the visual processing network, or its validity is limited to the areas processing faces is open and requires further studies. The above described differences in the capacity of PC explaining RS led to the following question: are there several neuronal mechanisms underlying the effect of P(rep) in the different cortical areas or are there other crucial factors determining these differences? One possible factor could be the level of expertise or prior experience with the given stimulus category. In fact, the results of Grotheer and Kovács (2014) suggest that expertise influences the magnitude of P(rep) modulation effect on RS, in a way that expectation effects only occur for familiar (real Roman characters) but not novel objects (false Roman characters). Since we assume that we are experts on faces, this could be a possible explanation for the different results. However, a more recent fMRIa study using face stimuli (Olkkonen et al., 2017) could show that expectation facilitates recognition behaviorally, but these modulatory effects could not be found in the BOLD signal of face-sensitive regions. Also, a recent study (Vinken et al., 2018) tested the effects of repetition probability in RS of the macaque inferotemporal cortex and found no interaction of P(rep) and RS on the spiking activity even though repetitions were task-relevant and repetition probability affected behavioral decisions. Again, in the current study, the sensory stimuli were of high expertise, i.e., faces. Therefore, future experiments will be needed in order to clearly understand whether the time window between cue and target stimuli lead to similar expectation effects for novel and familiar stimuli equally.

Furthermore, Larsson and Smith (2012) showed that perceptual expectation requires attention, specifically the P(rep) modulation effect on RS was only present if participants’ attention was directed toward the stimuli. Note that the experimental design of Larsson and Smith (2012) study focused on probabilistic, implicit expectations. Hence, it would be worthwhile to manipulate the subjects’ spatial and/or object-based attention, repetitions and expectations orthogonally, possibly applying a paradigm similar to Todorovic and de Lange (2012) or Grotheer and Kovács (2015). It is likely that the attentional state of the participants and therefore the applied task also plays a role in the fact that different results were obtained in the previous studies.

However, as it has been mentioned above, Kovács et al. (2013) did not observe P(rep) effects for every-day objects and the participants performed the same task (i.e., to signal the occurrence of a target trial, where the size difference between S1 and S2 was 55% by pressing a button) as in those prior studies, which reported significant P(rep) effects on RS for faces (Kovács et al., 2012; Grotheer et al., 2014). These findings suggest that differences in the attentional state alone are unlikely to induce such dissimilar perceptual expectation effects unless the given stimuli attract different attentional resources *per se*. In fact, there is evidence that faces recruit more attention than inanimate objects (Mack et al., 2002; New et al., 2007), which might explain the differences in P(rep) modulation effects on RS previously observed (Summerfield et al., 2008; Kovács et al., 2013). Still, to the best of our knowledge, there is no evidence that real Roman characters draw more attention than false Roman characters. Also, Olkkonen et al. (2017) could not find a P(rep) modulation effect on RS magnitude even though they used faces. Still, in this case, attentional effects could have caused the differences between the behavioral and neuroimaging data, as for the behavioral experiment attention was drawn to the images, whereas in the fMRI experiment participants performed an orthogonal task on target trials. Even though attention cannot be completely ruled out to explain previous findings, it is very unlikely as the source of the stability in terms of the ISI of expectation effect observed in the current study.

Importantly, in the current study there is a methodological asymmetry between the two expected experimental conditions, i.e., expected repetition (E_Rep) and the expected alternation (E_Alt). In the expected alternation condition the participants can only predict that the S2 is a previously unseen face, while in the expected repetition condition the predictions are that the S2 face is equal to S1. In other words, in the expected alternation condition participants can predict what the stimulus is not, but not what it is. Note that predictive theories argue that prediction updates occur repeatedly, and beliefs are gradually refined until the sensory system settles on the most likely interpretation of the inputs. Considering this, one can reason that if the statistical regularities of an environment are against our “default” predictions (i.e., learned based on experience), the strength of those predictions would be continuously diminished, due to constant updates. There is, therefore, a gap in the precision level of the predictions created in these two conditions. Still, there is an expectation effect on the alternation trials for both Immediate and *Delayed* ISI lengths (see Figure 3), in a way that the BOLD response is larger for surprising alternation than for the expected one. Following this line of thought, one question arises: what would happen if the alternation is predictable? Pajani et al. (2017) employed the influential Summerfield paradigm (2008) with an additional alternating block type where alternations were predictable. Participants could predict the S2 face based on S1, as S2 was specifically paired with S1. The participants were not aware of the contingencies but learned those in a preceding training session on the day before the scanning session. The authors found that predictable alternation trials elicited reduced FFA responses, as compared to unpredictable faces. Interestingly, repetition trials showed similar neuronal activation when presented in alternation blocks to when presented in the predictable alternation blocks. In other words, even though these repetition trials are surprising FFA responses were more suppressed than for predictable. In fact, repetition is always expected as it is the default expectation and, therefore, even with the alternating trials being predictable and expected, default expectations of repetition maintain and are stronger than the experimentally induced perceptually expectations. Still, it is not yet known how the predictable alternation affects cue-based, explicit expectations. Thus, future training studies will be necessary.

The gender-identification task used in the current study requires attention to S1 and not to S2 (Todorovic and de Lange, 2012), which can lead to different attentional states between S1 and S2. Prior studies using this task (Grotheer and Kovács, 2015; Amado et al., 2016) revealed that subjects utilized different repetition probabilities to perform the task. In other words, even if they did not know or remember the gender of the first face, they could expect the faces to be repeated or alternating, congruent on the gender of S1. If so, then participants should show perfect performance and faster reaction times for expected trials. Interestingly, the results of the current study do not show any behavioral facilitation response for expected trials, a result similar to those of Todorovic and de Lange (2012). One possible explanation is that the gender-identification of S1 is less dependent on the effects of expectation and surprise than that of S2. We decided the behavioral task relied on the discrimination of the S1 gender, to make sure participants directed their attention to the S1 in a way that expectations were induced and to ensure those expectations were not incorrect due to wrong discrimination of the S1 gender. It is possible that the chosen behavioral task might have directed attention toward S1 and way from S2, still, we found RS and ES effects in the FFA. A very recent study (Olkkonen et al., 2017), on the other hand, found a strong behavioral modulation of a priming effect (shorter RT for repeated as compared to alternating trials) depending on the likelihood of repetitions (larger modulation for expected as compared to unexpected trials). Surprisingly and contrary to many prior studies, the fMRIa was not modulated by expectation in this study, suggesting the relative independence of behavioral and neuroimaging correlates of expectation and urging further experiments, testing the issue.

### Possible ISI Variability Effects

In addition, we also know that the frequency or pace of events is a crucial parameter for the creation of expectations. A central timing system also referred to as “pacemaker,” describes that the pace/frequency of the event occurrences enables the creation of temporal perception units (Zakay and Block, 1997). Furthermore, it has been proposed that these local temporal perception units feed information to central systems (Matthews, 2015) and probably have an important role in prediction generation as well. Summerfield et al. (2011) investigated in an electrophysiological study how the consistency of stimulus repetition influences the effects of expectation and RS using stable (30–40 trial long) and volatile (10 trial long) blocks of stimulus presentations. Note that expectation was manipulated using different repetition probabilities in these blocks. Their results showed that RS was modulated by expectations at central electrodes for the stable, long blocks, while no modulation was present for the volatile, shorter blocks. As stability over time (sometimes labeled as “time-variability,” see Friston, 2010) can play a role in forming expectations, it will be important to test possible effects of ISI variability and the ISI length, independently. This relates to the major limitation of the current experiment, i.e., the fact that the two ISIs had different variability characteristics, as we, due to methodological constraints, only had one long ISI but variable and one short and at the same time constant ISI condition. Furthermore, the possibility that activation differences found between the *Immediate* and the *Delayed* ISIs are dependent on the different synchrony levels cannot be excluded as the additionally longer ITIs we used for the *Delayed* condition might also contribute to different overall temporal patterns. Therefore, further experiments are necessary to disentangle these two distinct effects (variability and length).

### Whole-Brain Analysis

The results obtained by the whole-brain analysis are in line with the previous studies that propose different neuronal mechanisms for short and long lagged cue-target stimulation periods. The results show several brain activation differences between the *Immediate* and the *Delayed* ISIs. Yet no significant differences between these two conditions were found in the FFA. Moreover, the whole-brain analysis did not elicit main effects of trial or expectation conditions which were previously found by Grotheer and Kovács (2015), Amado et al. (2016). The lack of these effects in the present study might be due to the lower number of trials in comparison with the former studies. Furthermore, the possibility that activation differences found between the *Immediate* and the *Delayed* ISIs are dependent on the different variability levels (constant and variable) cannot be excluded.

## Conclusion

In conclusion, this study shows that RS and expectation effects in the FFA are independent and additive processes for both *Immediate* and *Delayed* ISIs. As no significant difference was found between the two ISI lengths in the FFA, we can conclude the effects of repetition and expectation are maintained for several seconds in the FFA.

## Data Availability Statement

The datasets generated for this study are available on request to the corresponding author.

## Ethics Statement

The studies involving human participants were reviewed and approved by the Ethics Committee of the Faculty of Social and Behavioural Sciences of the University of Jena. The patients/participants provided their written informed consent to participate in this study. Written informed consent was obtained from the individual(s) for the publication of any potentially identifiable images or data included in this manuscript.

## Author Contributions

CA, MG, and GK designed the concept of the manuscript. MG and NW ran the experiments and analyzed the data together with CA. CA, S-MR, and GK wrote the manuscript.

## Conflict of Interest

The authors declare that the research was conducted in the absence of any commercial or financial relationships that could be construed as a potential conflict of interest.
